# CAS9 is a genome mutator by directly disrupting DNA-PK dependent DNA repair pathway

**DOI:** 10.1007/s13238-020-00699-6

**Published:** 2020-03-13

**Authors:** Shuxiang Xu, Jinchul Kim, Qingshuang Tang, Qu Chen, Jingfeng Liu, Yang Xu, Xuemei Fu

**Affiliations:** 1 Cancer Research Institute, Guangdong Provincial Key Laboratory of Cancer Immunotherapy, School of Basic Medical Sciences, Southern Medical University, Guangzhou, 510515 China; 2 The Eighth Affiliated Hospital, Sun Yat-sen University, Shenzhen, 518033 China; 3 Division of Biological Sciences, University of California, San Diego, 9500 Gilman Drive, La Jolla, CA 92093 USA; 4 Shenzhen Children’s Hospital, Shenzhen, 518026 China

**Keywords:** CAS9, DNA-PK, DNA double-stranded breaks, genetic instability, DNA repair

## Abstract

With its high efficiency for site-specific genome editing and easy manipulation, the clustered regularly interspaced short palindromic repeats (CRISPR)/ CRISPR associated protein 9 (CAS9) system has become the most widely used gene editing technology in biomedical research. In addition, significant progress has been made for the clinical development of CRISPR/CAS9 based gene therapies of human diseases, several of which are entering clinical trials. Here we report that CAS9 protein can function as a genome mutator independent of any exogenous guide RNA (gRNA) in human cells, promoting genomic DNA double-stranded break (DSB) damage and genomic instability. CAS9 interacts with the KU86 subunit of the DNA-dependent protein kinase (DNA-PK) complex and disrupts the interaction between KU86 and its kinase subunit, leading to defective DNA-PK-dependent repair of DNA DSB damage via non-homologous end-joining (NHEJ) pathway. XCAS9 is a CAS9 variant with potentially higher fidelity and broader compatibility, and dCAS9 is a CAS9 variant without nuclease activity. We show that XCAS9 and dCAS9 also interact with KU86 and disrupt DNA DSB repair. Considering the critical roles of DNA-PK in maintaining genomic stability and the pleiotropic impact of DNA DSB damage responses on cellular proliferation and survival, our findings caution the interpretation of data involving CRISPR/CAS9-based gene editing and raise serious safety concerns of CRISPR/CAS9 system in clinical application.

## Introduction

CAS9 is a type II DNA endonuclease widely used in genome editing and epigenetic modification (Jinek et al., 2012; Cong et al., 2013; Mali et al., 2013). CAS9 can efficiently target the entire genomic DNA sequence using the Protospacer Adjacent Motif (PAM) domain and a guide RNA (gRNA) (Komor et al., 2017; Murovec et al., 2017). In addition to its extensive use in biomedical research, the clinical application of the CRISPR/CAS9 system in human therapy has been intensively investigated (Barrangou and Doudna, 2016; Dever et al., 2016; Urnov, 2018; WareJoncas et al., 2018; Zhu et al., 2019). Variants of CAS9 have also been developed for distinct biological functions and improved editing fidelity. For example, the nuclease dead-CAS9 (dCAS9) has been developed for gene activation (Gilbert et al., 2013; Maeder et al., 2013), RNA editing (Cpf1) (Zetsche et al., 2015), and some smaller editing systems (Harrington et al., 2018). However, a number of problems still challenge the broad utility of CAS9 in research and clinical application (Tan et al., 2015; Haapaniemi et al., 2018; Ihry et al., 2018). For example, recent reports indicate that the combination of CAS9 and gRNA introduces genomic DNA DSBs, leading to p53-dependent cytotoxicity of human pluripotent stem cells (Ihry et al., 2018). Using long-read sequencing, researchers recently show that the genome editing mediated by CRISPR/CAS9 and gRNA could lead to large DNA deletions and complex rearrangements in the genome (Guo et al., 2018; Kosicki et al., 2018; Lei et al., 2018).

DNA double-stand break (DSB) damage is the most common genomic DNA lesion during cellular proliferation and genome editing (Jackson and Bartek, 2009), which could be repaired by two pathways: the direct DNA ligation of the broken DNA ends termed NHEJ and homologous DNA template guided homologous directed repair (HDR) (Mladenov and Iliakis, 2011). Immediately after the introduction of DSB, the DNA repair pathways are activated with a large panel of proteins recruited to the site of DNA DSBs (Mladenov and Iliakis, 2011). In the context of NHEJ, Ku70/86 heterodimer bind to the DNA DSBs to stabilize the DNA DSB ends and then recruit DNA dependent protein kinase catalytic subunit (DNA-PKcs) to the site of DNA DSB (Davis et al., 2014). DNA-PK complex is required for NHEJ by recruiting and phosphorylating a panel of the DNA repair factors including XRCC4 to facilitate DNA DSB repair (Uematsu et al., 2007; Davis et al., 2014). We demonstrate that CAS9 can disrupt the formation of DNA-PK complex through the interaction with KU86, promoting DNA DSB damage.

## Results and discussion

### CAS9 induces DNA DSB damage in human cells independent of gRNA

**Figure 1:**
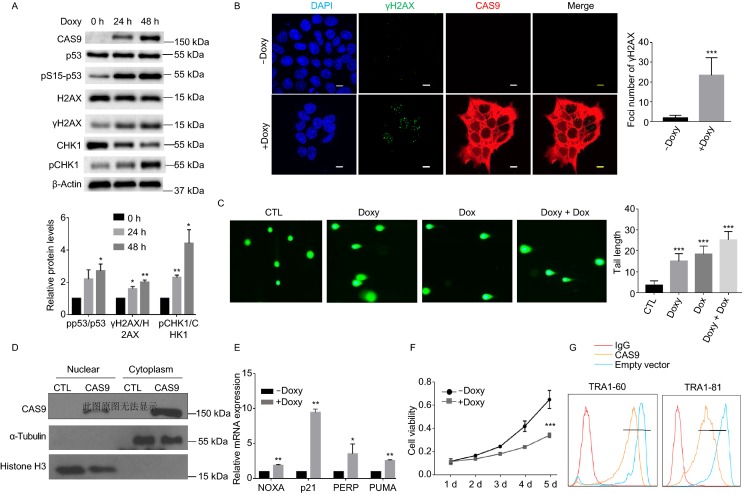
**The expression of CAS9 alone in hESCs promotes DNA DSB damage and activates DNA damage response pathway**. (A) Inducible expression of CAS9 in hESCs activated DNA damage response. Lentivirus harboring the inducible CAS9 expression cassette or control empty vector was used to transduce hESCs. The expression of CAS9 was induced with 2 µg/mL doxycycline (Doxy) treatment for 0, 24 h and 48 h. The relative levels of the phosphorylation of p53, H2AX, CHK1 were indicated at the bottom. *n* = 3. Data are presented as mean value ± SD. *P < 0.05, **P < 0.01. (B) The expression of CAS9 induced the number of the γH2AX foci in hESC. hESCs with CAS9 inducible expression cassette were plated on chamber slides and treated with or without 2 µg/mL doxycycline for 3 days. The γH2AX foci were detected by a confocal microscope. Scale bar, 10 µm. Unpaired t test. *n* = 20. Data are presented as mean value ± SD. ***P < 0.001. (C) Detection of DNA DSB damage by Comet assay in hESCs after various treatments. CTL, hESCs with empty expression vector treated with 2 µg/mL doxycycline (Doxy), doxorubicin (Dox), Doxy + Dox, hESCs with CAS9 inducible expression vector were treated with 2 µg/mL doxycycline for three days, 0.5 µmol/L Dox for 2 h, 2 µg/mL doxycycline for three days + 0.5µmol/L Dox for 2 h. Unpaired *t* test. *n* = 20. Data are presented as mean value ± SD. ***P < 0.001. (D) CAS9 is present in both nucleus and cytoplasm. CTL or CAS9, hESCs with lentiviral empty vector or CAS9 inducible expression cassette were treated with 2 µg/mL doxycycline for 3 days. Cells were fractionated into nuclear and cytoplasmic fractions, and examined for the levels of CAS9 as well as nuclear and cytoplasmic proteins histone H3 and α-tubulin. (E) The expression of p53 target genes in hESCs after the expression of CAS9. CTL, CAS9, hESCs with CAS9 inducible expression vector were treated with or without 2 µg/mL doxycycline for 3 days. *n* = 3. Data are presented as mean value ± SD. *P < 0.05, **P < 0.01, ***P < 0.001. (F) Cellular proliferation after the expression of CAS9 in hESCs. *n* = 3. Data are presented as mean value ± SD. ***P < 0.001. (G) The impact of CAS9 expression on the pluripotency of hESCs. The pluripotency makers TRA1-60 and TRA1-81 were analyzed in hESCs with or without CAS9 expression induced by 2 µg/mL Doxy for 4 days

For the clinical application of any gene editing technology, the genetically modified cells must maintain genomic stability. Because there is no reported study to investigate the impact of CAS9 on the genomic stability in the absence of gRNA, we used the doxycycline (Doxy) inducible expression system to induce the expression of CAS9 in human embryonic stem cells (hESCs). The expression of CAS9 protein in the absence of any exogenous gRNA activated DNA damage responses, including the phosphorylation of p53 at Ser15, CHK1 at Ser317, and H2AX at Ser139 (Fig. 1A). Consistent with this finding, the expression of CAS9 protein alone in hESCs was sufficient to induce the foci formation of H2AX at Ser139 (γH2AX), a marker for DNA DSBs in the genome (Fig. 1B). We used comet assay to confirm that the expression of CAS9 protein alone in hESCs induced DNA DSB damage (Fig. 1C). Nuclear fractionation analysis indicated that CAS9 is present in both nucleus and cytoplasm (Fig. 1D). Activation of p53 in hESCs can activate apoptosis and differentiation of hESCs as previously shown (Lin et al., 2005; Song et al., 2010). The DNA DSB damage induced by CAS9 activated the expression of p53 target genes such as *PUMA*, *p21*, *PERP* and *NOXA*, leading to the cell death and differentiation of hESCs (Fig. 1E–G).

**Figure 2:**
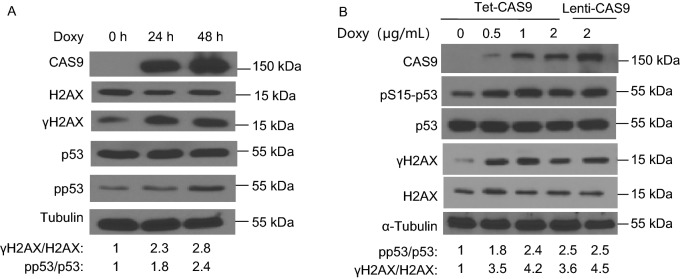
**The expression of CAS9 in hiPSCs and hESCs promotes DNA DSB damage**. (A) The inducible expression of CAS9 promotes DNA DSB damage responses in hiPSCs after 2 µg/mL Doxy treatment. The relative levels of the phosphorylation of p53 and H2AX are indicated at the bottom. Consistent data were obtained from two independent experiments. (B) The impact of expression levels of CAS9 on DNA DSB damage in hESCs. At the same lentiviral titers, the expression levels of CAS9 in hESCs transduced by standard lentiviral vector are higher than those transduced by the inducible lentiviral vector after 2 µg/mL Doxy treatment. Much lower expression levels of CAS9 can also promote DNA DSB damage in hESCs after the treatment with lower dosages of Doxy. The relative levels of the phosphorylation of p53 and H2AX are indicated at the bottom

**Figure 3:**
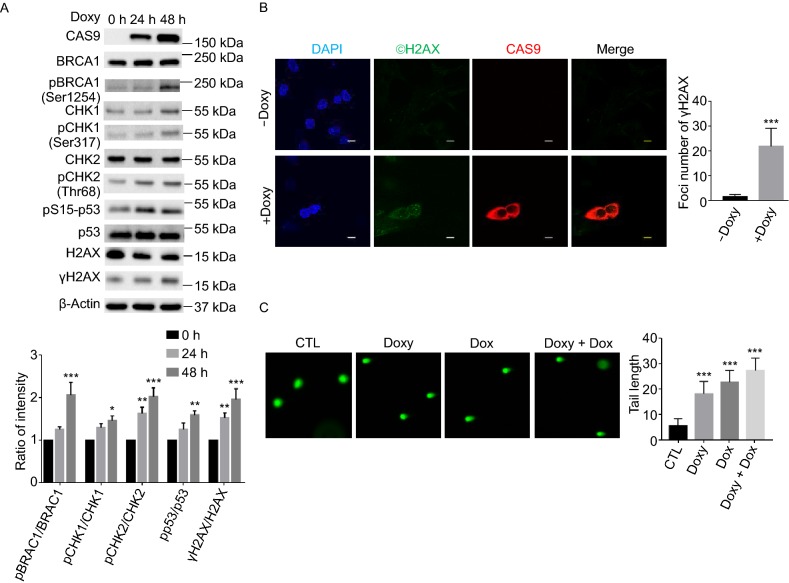
**The expression of CAS9 in human fibroblasts promotes DNA DSB damage and activates DNA damage response pathways**. (A) The expression of CAS9 in human fibroblasts activates DNA damage responses. The expression of CAS9 was induced with 2 µg/mL Doxy treatment. The relative levels of phosphorylation of BRCA1, CHK1, CHK2 and p53 are indicated at the bottom. *n* = 3. Data are presented as mean value ± SD. *P < 0.05, **P < 0.01, ***P < 0.001. (B) The expression of CAS9 increased the number of γH2AX foci in human fibroblasts. CTL or CAS9, human fibroblasts with CAS9 inducible expression cassette plated on chamber slides were treated with or without 2 µg/mL doxyc ycline for 3 days. The expression of CAS9 and γH2AX foci was revealed by immunoflourescence analysis. Representative images are shown. Scale bar, 10 µm. Unpaired *t* test. *n* = 20. Data are presented as mean values ± SD. ***P < 0.001. (C) CAS9 induces DNA DSB damage in human fibroblasts. CTL, human fibroblasts with lentiviral empty vector were treated with 2 µg/mL doxycycline for three days; Doxy, Dox, Doxy + Dox, human fibroblasts with lentiviral CAS9 inducible expression vector were treated with 2 µg/mL doxycycline for 3 days or 0.5 µmol/L Dox for 2 h or 2 µg/mL doxycycline for three days + 0.5 µmol/L Dox for 2 h, respectively. Representative images are shown. *n* = 40. Unpaired t test. Data are presented as mean value ± SD. **P < 0.01

To confirm this genome mutator function of CAS9 in mammalian cells, we employed the same CAS9 inducible expression system to express CAS9 in human induced pluripotent stem cells (hiPSCs) and human fibroblasts. Consistent with the findings in hESCs, the expression of CAS9 alone in hiPSCs and human fibroblasts was sufficient to induce DNA DSB damage and activate DNA damage responses (Figs. 2A and 3A–C). Using decreasing dosages of Doxy to induce much lower expression of CAS9, we showed that, at the expression levels much lower than those of standard lenti-viral transduction, CAS9 could still induce DNA DSB damage (Fig. 2B). Therefore, CAS9 can induce DNA DSB damage in mammalian cells independently of exogenous gRNA.

### CAS9 disrupts DNA-PK complex by interacting with KU86

**Figure 4:**
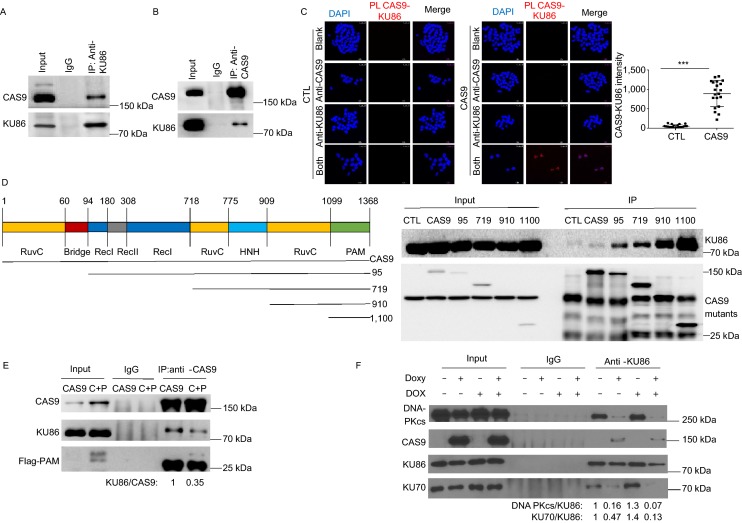
**CAS9 interacts with KU86 and disrupts the formation of DNA-PK**. (A and B) Reciprocal immunoprecipitation shows the interaction between CAS9 and KU86. Protein extracts from hESCs expressing CAS9 were immunoprecipitated with anti-KU86 (A) or anti-CAS9 (B), and immune precipitates were analyzed for the presence of KU86 and CAS9. (C) The interaction between CAS9 and KU86 was confirmed by the proximity ligation analysis (PLA). Cell nucleus were revealed by DAPI (Blue) staining and the CAS9-KU86 interaction indicated by red color. Scale bar, 25 µm. Unpaired *t* test. *n* = 20. Data are presented as mean value ± SD. ***P < 0.001. (D) Mapping the domain of CAS9 involved in the interaction with KU86. The Flag-tagged deletional mutants of CAS9 expressed in 293FT cells were immunoprecipitated with anti-flag antibody. Immune precipitates were analyzed for the presence of CAS9 mutants and KU86. (E) The expression of the PAM domain (1100) of CAS9 disrupted the interaction between CAS9 and KU86. The levels of CAS9, KU86, PAM in the input and immunoprecipitate were analyzed by Western blot. The ratio of CAS9 versus KU86 in the immunoprecipitate is shown at the bottom. (F) CAS9 disrupts the formation of DNA-PK complex. Protein extracts of cells in the presence and absence of CAS9 and DOX treatment were immunoprecipitated with anti-KU86 antibody. The levels of KU70, DNA-PKcs and CAS9 in the immunoprecipitate were analyzed. The relative ratios of DNA-PKcs versus KU86 or KU70 versus KU86 are indicated

To elucidate the mechanism how CAS9 induces DNA DSB damage, we investigated the potential interaction between CAS9 and the components of DNA repair pathways. We discovered that CAS9 interacted with KU86, a component of DNA-PK complex essential for NHEJ pathway (Fig. 4A and 4B). The interaction between CAS9 and KU86 was further confirmed by proximity ligation assay (Fig. 4C). Using a series of deletional mutants of CAS9, we found that the PAM domain of CAS9 was involved in the interaction with KU86 and other domains of CAS9 might interfere with the interaction between the PAM domain and KU86 (Fig. 4D). In further support of this notion, the expression of the PAM domain of CAS9 reduced the interaction between CAS9 and KU86 (Fig. 4E). The expression of CAS9 disrupted the interaction between KU86 with DNA-PKcs or K70, indicating that the interaction between CAS9 and KU86 inhibited the formation of DNA-PK complex (Fig. 4F).

**Figure 5:**
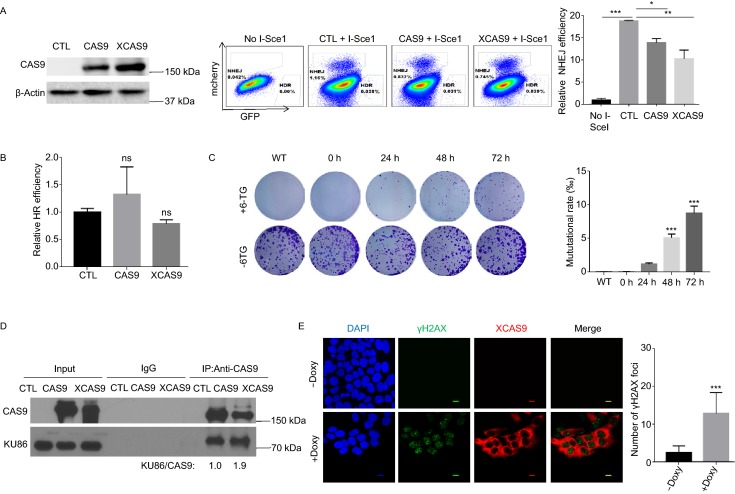
**Both CAS9 and XCAS9 impair NHEJ and induce genetic mutations**. (A and B) Expression of CAS9 and XCAS9 impairs NHEJ. Traffic Light Reporter system was established in 293 cells harboring the CAS9 and XCAS9 inducible expression vectors. After the induction of CAS9 and XCAS9 expression with 2 µg/mL doxycycline for 3 days (left panel), the efficiency of NHEJ (mcherry) and HDR (GFP) was analyzed by flow cytometry (middle panel). Statistic analysis of the efficiency of NHEJ (left panel of A) and HDR (B) is presented. *n* = 3. Data are presented as mean values ± SD. *P < 0.05, **P < 0.01, ***P < 0.001. ns, non-significant. (C) The expression of CAS9 in hESCs induces genomic mutations at the endogenous HPRT locus. After hESCs harboring CAS9 inducible expression vector were selected with HAT medium for 5 days, they were treated with 2 µg/mL doxycycline for CAS9 expression for various time periods, and subsequently, treated with 5 µg/mL 6-TG or mock treated for 4 days. Mutational rate is calculated as the ratio of colony number in 6-TG treated samples versus untreated controls. *n* = 3. Data are presented as mean values ± SD. ***P < 0.001. (D) XCAS9 interacts with KU86. Protein extracts from 293FT cells expressing Flag-tagged CAS9 or XCAS9 were immunoprecipitated with anti-Flag antibody. The immune precipitates were analyzed for the presence of CAS9, XCAS9 and KU86. The relative ratio of KU86 versus CAS9 or XCAS9 is indicated. (E) The expression of XCAS9 increases the number of γH2AX foci in hESCs. hESCs harboring XCAS9 inducible expression vector were treated with or without 2 µg/mL doxycycline for 3 days. *n* = 20. Scale bar, 10 µm. Data are presented as mean values ± SD. ***P < 0.01

The formation of DNA-PK complex at the site of DNA DSB is required for repairing DNA DSB via NHEJ and maintaining genomic stability (Davis et al., 2014). Therefore, we hypothesized that CAS9 could inhibit DNA DSB repair via NHEJ pathway and induce genomic instability by disrupting the formation of DNA-PK complex. In support of this notion, the expression of CAS9 in hESCs reduced the efficiency to repair DNA DSB damage via NHEJ pathway (Fig. 5A), but did not affect the efficiency to repair DNA DSB damage via HDR pathway (Fig. 5B). In addition, the expression of CAS9 in hESCs significantly increased the rate of spontaneous mutation of the HPRT gene in hESCs, indicating that CAS9 induces genomic instability (Fig. 5C). Therefore, the expression of CAS9 induces genomic instability in hESCs.

### High fidelity or nuclease-dead CAS9 variants and Cpf1 induce DNA DSB damage

In an attempt to improve the fidelity of CAS9 in gene editing, recent studies have described CAS9 variants such as xCAS9 that appear to have higher fidelity (Hu et al., 2018). However, similarly to CAS9, XCAS9 also interacted with KU86 and induced DNA DSB damage independently of the exogenous gRNA (Fig. 5D and 5E). In addition, XCAS9 impaired DNA DSB repair via NHEJ but did not affect the DNA DSB repair via HDR (Fig. 5A). Therefore, XCAS9 is also a genome mutator that can promote DNA DSB damage in the absence of any gRNAs.

**Figure 6:**
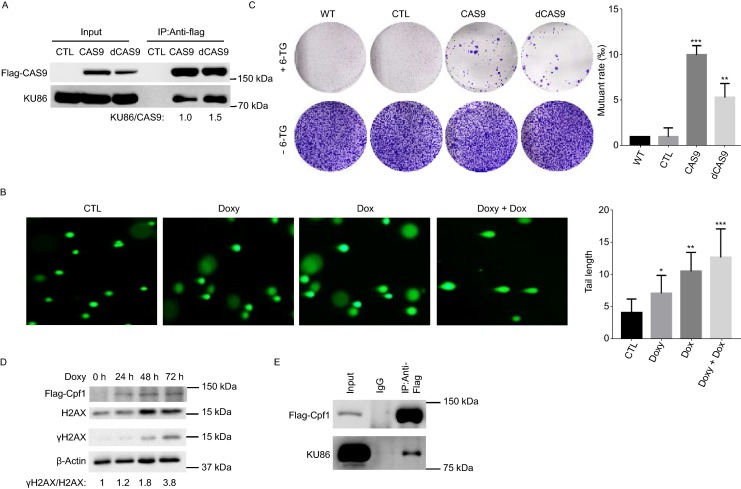
**dCAS9 and Cpf1 impair NHEJ and induce genetic mutations**. (A) Co-immunoprecipitation assay confirmed the interaction between dCAS9 and KU86. (B) Comet assay analysis of DNA damage in hESCs expressing dCAS9 or treated with doxorubicin. CTL, human fibroblasts with lentiviral empty vector were treated with 2 µg/mL doxycycline for three days; Doxy, Dox, Doxy + Dox, human fibroblasts with lentiviral CAS9 inducible expression vector were treated with 2 µg/mL doxycycline for 3 days or 0.5 µmol/L Dox for 2 h or 2 µg/mL doxycycline for three days + 0.5 µmol/L Dox for 2 h, respectively. Tail length was analyzed using Image J software. Data are represented as mean ± SD. *P < 0.05, **P < 0.01, ***P < 0.001. (C) The expression of dCAS9 induces mutation of endogenous HPRT gene. WT, WT hESCs; CTL, CAS9, dCAS9, hESCs with empty expression vector, CAS9 inducible expression vector. Cells with dCAS9 inducible expression vector were treated with 2 µg/mL doxycycline for 3 days before HAT treatment. *n* = 3. Data are presented as mean value ± SD. **P < 0.01, ***P < 0.001. (D) The expression of Cpf1 increased the levels of γH2AX. (E) Cpf1 interacts with KU86 as confirmed by Co-immunoprecipitation. Protein extract of Flag-tagged Cpf1 was immunoprecipitated with anti-Flag antibody and the presence of Cpf1 and KU86 in the immunoprecipitate was examined by Western blot

Mammalian cells might express endogenous gRNA-like small RNA that could work with CAS9 to induce DNA damage. To test this possibility, we examined the impact of the expression of dCAS9, which is defective in the nuclease activity (Gilbert et al., 2013; Maeder et al., 2013), on DNA DSB damage. Similar to CAS9 and XCAS9, dCAS9 also interacted with KU86 (Fig. 6A). In addition, the expression of dCAS9 in hESCs also promoted DNA DSB damage and genetic mutations at HPRT locus (Fig. 6B and 6C). While the impact of dCAS9 on DNA damage appeared to be less dramatic than CAS9, the results demonstrate that CAS9 can induce DNA damage independent of its nuclease activity. Similarly to CAS9, Cpf1 also interacted with KU86 and activated DNA DSB damage (Fig. 6D and 6E).

Previous studies evaluated the genomic stability of cells after the site-specific gene editing induced by CAS9 + gRNA, suggesting that the specificity of gRNA and the delivery method of CAS9/gRNA could be optimized to improve the fidelity and safety of this gene editing technology (Haapaniemi et al., 2018; Ihry et al., 2018; Kosicki et al., 2018). Our data demonstrate that CAS9 and its high-fidelity variant XCAS9 are genome mutators by promoting DNA DSB damage and genetic mutations independently of gRNA. This intrinsic oncogenic activity of CAS9 is achieved by disrupting DNA-PK complex, leading to impaired DNA DSB repair via NHEJ pathway. In addition, it has been well documented that defects in the DNA-PK function will promote DNA DSB damage and genetic instability (Davis et al., 2014). Therefore, our findings raise concerns for the safety of CRISPR/CAS9 system based human therapy. The development of CAS9 variants that retain the gene editing activity of wild type CAS9 but do not disrupt DNA-PK activity might help to improve the fidelity of gene editing technology and safety of the CRISPR/CAS9 system.

## Materials and methods

### Cell lines, lentivirus production and transduction

Human embryonic stem cell line H9 was obtained from WiCell Research Institute, Inc. and cultured with mTeSR1 medium (STEMCELL, USA). Human fibroblasts were used between passages 3 to 12. The HEK 293 FT and HEK 293T cell lines were purchased from Thermo Scientific. Human fibroblasts and HEK 293 FT were cultured in DMEM (Gibco) supplemented with 10% fetal bovine serum (FBS) (Hyclone) and 1% penicillin-streptomycin (Pen/Strep) (Gibco) at 37 °C with 5% CO_2_. The lentivirus production and transduction were performed as we previously described (Kim et al., 2019). All cell lines were routinely checked for mycoplasma by a PCR detection kit.

### Expression vector construction

To construct vectors that express genes inducibly, CAS9, XCAS9, dCAS9 or Cpf1 cDNA was cloned into TetO-Fuw-PGK-Puro vector modified from TetO-Fuw-OSKM vector (Addgene plasmid 20321) linearized by EcoRI/AgeI digestion using Gibson Assembly (2× NEBuilder HiFi DNA Assembly Master Mix) as previously described (Kim et al., 2019). To generate KU86 and CAS9 deletional mutants, pLenti-CMV-GFP vector (Addgene 17448) was linearized by BamHI/SalI digestion and ligated with the PCR products of KU86 and different domains of CAS9 by Gibson assembly. The PCR primers were provided in Table 1.

**Table 1:** Primers used in this study.

	Sequence (5′-3′)
Primers for cloning	
Fuw-teto-CAS9/XCAS9/dCAS9-pgk-puro Gibson F	TATCGATAAGCTTGATATCGAATTCTCAGGCACCGGGCTTGCGGG
Fuw-teto-CAS9/XCAS9/dCAS9-pgk-puro Gibson R	ATCCAGCCTCCGCGGCCCCGAATTCGCCACCATGGACAAGAAGTACAGCAT
Fuw-teto-control-pgk-puro F	AATTCGCCACCATGGATTACAAAGACGATGACGATAAGTAGA
Fuw-teto-control-pgk-puro R	CCGGTCTACTTATCGTCATCGTCTTTGTAATCCATGGTGGCG
plenti-CAS9-pgk-puro Gibson F	ACCGACTCTAGAGGATCCGCCACCATGGACAAGAAGTACAGCATCGGCC
plenti-CAS9-pgk-puro Gibson R	TCCAGAGGTTGATTGTCGACCTACTTATCGTCATCGTCTT
Fuw-teto-95-pgk-puro Gibson F	GACCGATCCAGCCTCCGCGGCCCCGAATTCGCCACCATGGACAGCTTCTTCCACAGACTG
Fuw-teto-95-pgk-puro Gibson R	TGGAAAAGGCGCAACCCCAAACCGGTCTACTTATCGTCATCGTCTTTGTAATC
plenti-719-pgk-puro Gibson F	GACCGATCCAGCCTCCGCGGCCCCGAATTCGCCACCATGAGCCTGCACGAGCACATT
Fuw-teto-719-pgk-puro Gibson R	TGGAAAAGGCGCAACCCCAAACCGGTCTACTTATCGTCATCGTCTTTGTAATC
Fuw-teto-910-pgk-puro Gibson F	GACCGATCCAGCCTCCGCGGCCCCGAATTCGCCACCATGGAACTGGATAAGGCCGGCTT
Fuw-teto-910-pgk-puro Gibson R	TGGAAAAGGCGCAACCCCAAACCGGTCTACTTATCGTCATCGTCTTTGTAATC
Fuw-teto-1100-pgk-puro Gibson F	GACCGATCCAGCCTCCGCGGCCCCGAATTCGCCACCATGGTGCAGACAGGCGGCTTCA
Fuw-teto-1100-pgk-puro Gibson R	TGGAAAAGGCGCAACCCCAAACCGGTCTACTTATCGTCATCGTCTTTGTAATC
plenti-control-pgk-puro F	GATCCGCCACCATGTACCCATACGACGTCCCAGACTACGCTTAGG
plenti-control-pgk-puro R	TCGACCTAAGCGTAGTCTGGGACGTCGTATGGGTACATGGTGGCG
plenti-Ku86-pgk-puro Gibson F	GACACCGACTCTAGAGGATCCGCCACCATGTACCCATACGACGTCCCAGACTACGCTATGGTGCGGTCGGGGAATAAG
plenti-Ku86-pgk-puro Gibson R	TCACAAATTTTGTAATCCAGAGGTTGATTGTCGACGAATTCCTATATCATGTCCAATAAATCGTCC
Fuw-teto-Cpf1-pgk-puro Gibson F	AAGCCCTCGAACTGTGTCATGGTGGCGAATTCGGGGCCGCGGAGGCTGGAT
Fuw-teto-Cpf1-pgk-puro Gibson R	AGACGATGACGATAAGTAGACCGGTTTGGGGTTGCGCCTTTTCCA
Primers for RT-qPCR	
Puma F	ACGACCTCAACGCACAGTACGA
Puma R	CCTAATTGGGCTCCATCTCGGG
P21 F	ACCTGGAGACTCTCAGGGTCG
P21 R	TTAGGGCTTCCTCTTGGAGAAGAT
Perp F	TCATCCTGTGCATCTGCTTC
Perp R	GGGTTATCGTGAAGCCTGAA
Noxa F	ACCAAGCCGGATTTGCGATT
Noxa R	ACTTGCACTTGTTCCTCGTGG
β-actin F	AGCGAGCATCCCCCAAAGTT
β-actin R	GGGCACGAAGGCTCATCATT

### Western blot analysis and Co-immunoprecipitation (Co-IP)

Cells were extracted for total proteins using lysis buffer containing Protease and Phosphatase Inhibitor Cocktail (Cell Signaling Technology CST). For nuclear protein extraction, nuclear and cytoplasm protein extraction kit (Thermo Fisher Scientific) was used following the instruction. Protein was separated on 6%–15% SDS PAGE and transferred to 0.45 µm nitrocellulose membranes (Merck Millipore USA), which were blocked with the blocking buffer (5% skim milk in TBS with 0.05% Tween 20) and incubated with primary antibodies at 4 °C overnight. The membranes were incubated with secondary antibodies at room temperature for 1 h and detected with Supersignal West Pico or Dura exposure buffer (Thermo Fisher Scientific). For Co-IP, the cells were collected and lysed with IP lysis buffer (Thermo Fisher Scientific) containing protease inhibitor cocktail (Thermo Fisher Scientific) on ice for 30 min. After the centrifugation at 12,000 ×*g* for 20 min at 4 °C, the supernatants were immunoprecipitated with antibodies followed by incubating with magnetic protein A/G beads (Pierce) for 4 h. For IP analysis of phosphorylated proteins, cells were collected in the IP lysis buffer containing Protease and Phosphatase Inhibitor Cocktail. The antibodies used were provided in Table 2.

**Table 2:** Antibodies used in this study

Antibody	Source and catalog
Anti-γH2AX	Cell signaling (9718S, 80312S)
Anti-H2AX	Cell signaling (7631S)
Anti-p53	Santa Cruze (sc-126)
Anti-pp53	Cell signaling (9286S)
Anti-CHK1	Cell signaling (2360S)
Anti-pCHK1	Cell signaling (12302S)
Anti-H3	Abcam (ab1971)
Anti-KU86	Santa Cruze (sc-5280), Abcam (ab119935), Cell signaling (2753s)
Anti-KU70	Abcam (ab92450)
Anti-DNA PKcs	Abcam (ab70250)
Anti-BRCA1	Cell signaling (14823S)
Anti-p-BRCA1	Cell signaling (9009S)
Anti-CHK2	Cell signaling (6334S)
Anti-pCHK2	Cell signaling (2197S)
Anti-CAS9	Cell signaling (14697S), Abcam (ab189380), Novus Biologicals (NBP2-52717)
	(NBP2-52717)
Anti-β-actin	Cell signaling (4970S)
Anti-Flag	Thermo Fisher (MA1-91878)
Anti-α-tubulin	Santa Cruze (sc-73242)

### Proximity ligation analysis

hESCs with inducible CAS9 expression vector were plated on chamber slides and treated with 2 µg/mL doxycycline for 2 days. After the treatment, the cells were washed with PBS twice and then fixed with 4% paraformaldehyde (PFA) for 15 min at room temperature. After washing with PBS three times, cells were permeabilized with 0.3% Triton X-100 in PBS for 10 min at room temperature. Then Duolink^®^ In Situ Red Starter Kit Mouse/Rabbit (Sigma, DUO92101) was used to reveal the interaction between CAS9 and KU86 as instructed by the manufacturer. The antibodies of different species (rabbit anti-CAS9 antibody, mouse anti-KU86 antibody) were used to detect the proteins. DAPI (blue) was used to stain the nucleus.

### Immunofluorescence staining

1 × 10^3^ H9 or fibroblast cells were seeded onto the chamber slides coated with matrigel (Corning) and treated with 2 µg/mL doxycycline for 2 days. After the treatment, cells were washed with PBS twice, and then fixed with 4% paraformaldehyde (PFA) for 15 min at room temperature. After washing with PBS three times, cells were permeabilized with 0.3% Triton X-100 in PBS for 10 min, blocked with 2% bovine serum albumin (BSA) in PBS for 1 h at room temperature, stained with anti-CAS9 antibody (Abcam, 1:100) and anti-γH2AX antibody (CST, 1:100) at 4 °C overnight, followed by simultaneous incubation with Alexa Fluor 568-conjugated anti-mouse secondary antibody and Alexa Fluor 488-conjugated anti-rabbit antibody for 1 hr at room temperature. Slides were mounted using VECTASHIED solution (Vector) with DAPI. The images were acquired as previously described (Chen et al., 2018).

### Comet assay analysis

Comet assay was carried out as we previously described (Xiong et al., 2015). Briefly, hESCs or fibroblasts plated on the matrigel-coated 6-well plates were treated with 2 µg/mL doxycycline for 2 days, followed with or without the treatment with 0.5 µmol/L doxorubicin for 4 h. Cells were harvested, washed with ice-cold PBS twice, resuspended at a density of 10^5^ cells/mL, mixed with agarose at a 1:10 ratio, spread onto 3-well Comet Assay slides, and kept in dark for 15 min at room temperature. Slides were immersed in chilled lysis solution for 15 min, washed with chilled TBE buffer three times and electrophoresed in chilled TBE (Tris-borate-EDTA) buffer for 15 min at 20 V. Slides were then fixed in 70% ethanol and dried, and DNA was labeled with Vista Green DNA Dye diluted with TBE buffer. Images were captured and analyzed using Image J software.

### RNA purification and quantitative PCR analysis

The RNA extraction and qPCR were performed as we previously described (Kim et al., 2019). The primers used for qPCR were listed in Table 1.

### Cell viability

hESC cells were digested with TripLe (Gibco) to prepare single cells. 4 × 10^3^ cells/well were plated onto each well of the 96-well plates with triplicate wells per sample. Cell Counting Kit (CCK8, DoJindo) was used to evaluate cell viability according to the manufacturer’s instructions.

### Detection of spontaneous mutations of the HPRT gene in hESCs

The detection of spontaneous mutations of the HPRT gene in hESCs was performed as previously described (Kang et al., 2002). Briefly, hESCs with empty vector or with CAS9 inducible expression vector were treated with 1× hypoxanthine-aminopterin-thymidine (HAT) medium for 5 days to eliminate hESCs with HPRT mutations. After replating, 2 µg/mL doxycycline was added to induce the expression of CAS9 for 24 h, 48 h or 72 h. Cells were harvested and plated on triplicate 6 wells at a density of 10^5^ cells/well. These cultures were mock treated or treated with 5 µg/mL 6-thioguanine (6-TG) for 3 days to select for hESCs lacking functional HPRT. Cultures were incubated for 8–10 days and colonies fixed with 4% paraformaldehyde (PFA) for 10 min at RT. After washing with PBS three times, colonies were visualized by staining with crystal violet. The number of colonies was counted using the Image J software.

### Detection of DNA DSB repair via NEHJ and HDR pathways

We used the Traffic Light Reporter system to evaluate the efficiency to repair DNA DSBs via NHEJ and HDR pathways as previously described (Gomez-Cabello et al., 2013). Briefly, HEK293FT cells were transduced with Traffic Light Reporter virus and 5 μg/mL polybrene. After 48 h, cells were selected with 1 μg/mL puromycin for 3 days. Surviving clone was picked, expanded and verified with PCR to confirm the integration of the reporter harboring an I-sce1 site. Reporter cells were transduced with the control or inducible CAS9 expression lentivirus. 6 × 10^4^ control or cells with inducible CAS9 expression vector were plated onto the triplicate wells of 48-well plates. After the induction of the expression of CAS9, the cells were transduced with I-SceI-T2A-IFP or control empty lentivirus. Ten days later, cells were collected and analyzed by flow cytometry. The results were analyzed by Flowjo software.

### Statistical analysis

Any statistical method was used to calculate group variation was not estimated before experiments. Statistical significance was assayed with GraphPad Prism. For comparing two groups, *t*-test was used. *P < 0.05, **P < 0.01, ***P < 0.001, ns means non-significant.
